# Associations between occupational stress, work–family imbalance, and harmful alcohol consumption among workers: A longitudinal study

**DOI:** 10.1002/pcn5.70177

**Published:** 2025-08-05

**Authors:** Kunio Maekubo, Yasuhiko Deguchi, Shinichi Iwasaki, Shohei Okura, Ayaka Matsunaga, Kohei Yamashita, Koki Inoue

**Affiliations:** ^1^ Department of Neuropsychiatry, Graduate School of Medicine Osaka Metropolitan University Osaka Japan

**Keywords:** harmful alcohol consumption, longitudinal study, occupational stress, worker, work–family imbalance

## Abstract

**Aim:**

This study aimed to longitudinally examine the effects of occupational stress and bidirectional work–family spillover on harmful alcohol consumption (HAC) among workers.

**Methods:**

We conducted online surveys in December 2020 and June 2021 among Japanese workers aged 20–65. The follow‐up survey targeted participants from the initial wave and yielded 824 responses. After excluding individuals with an Alcohol Use Disorders Identification Test (AUDIT) score of 8 or higher at baseline, the final analysis included 640 participants. The study measured HAC using AUDIT. Researchers assessed bidirectional work–family spillover (positive and negative) using the Japanese version of the Survey Work–Home Interaction—NijmeGen (SWING‐J). They measured occupational stress factors, including quantitative workload, job control, supervisor support, and coworker support, using the Japanese version of the Generic Job Stress Questionnaire (GJSQ). Participants were categorized into the HAC and non‐HAC groups based on follow‐up AUDIT scores. We conducted a logistic regression analysis using the stepwise method.

**Results:**

A total of 68 participants (10.6%) were classified into the HAC group, and 572 (89.4%) into the non‐HAC group. The logistic regression analysis showed that higher negative work‐to‐family spillover scores (OR = 1.10, 95% CI = 1.04–1.17) and lower quantitative workload scores (OR = 0.93, 95% CI = 0.89–0.98) are significantly associated with HAC.

**Conclusion:**

Understanding the causal relationships between occupational stress, work–family dynamics, and HAC can help inform more effective prevention strategies for problematic alcohol use among workers.

## INTRODUCTION

Harmful use of alcohol is a significant contributor to the global burden of disease and remains a critical public health concern. The Global Burden of Disease Study 2016 shows that alcohol consumption is the seventh leading risk factor for both mortality and disability‐adjusted life years (DALYs) worldwide. In that year, alcohol accounted for 6.8% of all deaths among men and 2.2% among women. Among individuals aged 15 to 49 years, who represent the most economically and socially active segment of the population, alcohol use is the single leading risk factor.^1^ In Japan, a national strategy to address alcohol‐related problems began in 2016 to strengthen prevention and support.^2^ In 2021, the approach expanded to include more targeted interventions that focus on the onset, progression, and recurrence of alcohol‐related disorders.^3^ From a preventive perspective, it is essential to longitudinally identify factors that influence drinking habits and predict future risk. Even among the working‐age population, problematic alcohol use receives significant attention due to its negative effect on productivity and occupational safety.^4^, ^5^, ^6^ Previous studies suggest that occupational stress and work−family imbalance may increase the risk of problematic drinking among workers.^7^, ^8^, ^9^, ^10^

Workplace stress contributes to various psychological and physical health issues, including depression, anxiety disorders, hypertension, and cardiovascular diseases. These conditions lead to reduced productivity and increased absenteeism.^11^ Specific workplace stressors, such as long working hours, excessive workloads, and low compensation, are associated with problematic alcohol use.^12^, ^13^ Social support in the workplace functions as a buffer against psychological distress,^14^, ^15^ and social support may also act as a protective factor against problematic drinking.^16^

Additionally, an imbalance between work and family roles may increase psychological stress. This stress could, in turn, lead to drinking as a coping behavior.^17^, ^18^ One important manifestation of work–family imbalance is role spillover. Role spillover refers to the transfer of stress or resources between work and family domains.^19^, ^20^ Work–family conflict, which this study conceptualizes as negative spillover, arises when the demands of work and family roles are incompatible. Researchers have associated work–family conflict with adverse health outcomes, such as increased burnout, depression, poor physical health, and heavy alcohol use.^21^, ^22^, ^23^ Conversely, work–family enrichment, which represents positive spillover, is linked to reduced depression and burnout, as well as greater life satisfaction.^19^, ^22^, ^23^, ^24^

Given these associations, understanding the relationship between occupational stress, work–family imbalance, and alcohol consumption may help reduce the risk of problematic drinking. However, most existing studies use cross‐sectional designs, and there is limited longitudinal evidence to support causal inferences.^25^, ^26^, ^27^, ^28^, ^29^ Therefore, this study uses a longitudinal design to clarify how occupational stress and work–life imbalance affect the onset of problematic drinking. To comprehensively understand the health implications of work–family dynamics, it is important to consider both the valence (positive or negative) and direction (work‐to‐family and family‐to‐work) of spillover.^30^ One study shows that positive spillover may suppress alcohol use.^31^ However, few studies examine both positive and negative spillover in relation to drinking behavior, and researchers have not identified any longitudinal studies. Thus, in this study, we examine both negative (conflict) and positive (enrichment) spillover as they relate to problematic drinking.

This study aims to investigate the associations between occupational stress, bidirectional work–family spillover, and harmful alcohol consumption (HAC) among workers. We hypothesize that higher quantitative workload and lower job control are associated with an increased risk of HAC, while social support functions as a protective factor. We also hypothesize that negative spillover in both directions, work‐to‐family and family‐to‐work, is positively associated with HAC. In contrast, positive spillover in both directions is inversely associated with HAC.

## METHODS

### Study design, participants, and procedure

We conducted a longitudinal online survey twice through Macromill, Inc. Japan, a Japanese research company. The first survey took place from December 16 to 17, 2020. The second survey occurred from June 16 to 21, 2021. Eligible participants included individuals residing in Japan who were currently employed and between the ages of 20 and 65. Researchers recruited a sample of approximately 1000 participants from the approximately 10 million individuals registered, targeting Japanese workers with diverse employment statuses. This sample size is considered sufficient for the present analysis. Participation in the survey was voluntary, and all participants received information about this. All participants provided informed consent through an online consent form. Researchers ensured that they could not access any personal information, such as names, phone numbers, or home addresses.

In the first survey, data were collected from 1070 workers. The second survey was administered to those who completed the first survey, and complete responses were obtained from 824 individuals. Of the 824 respondents who answered both surveys, participants who scored 8 points or higher on the Alcohol Use Disorders Identification Test (AUDIT) in the first survey are excluded (*n* = 184). The final 640 participants who met the exclusion criteria were analyzed. A cutoff score of 8 on AUDIT shows high sensitivity and acceptable specificity for ICD‐10 alcohol use disorders and future harmful risks in most studies.^32^, ^33^

This study follows the Declaration of Helsinki and its later amendments. The Ethics Committee of Osaka Metropolitan University approved the study design (Approval No. 4245). The research team stored all data exclusively in their database. Employers and affiliated institutions of the participants do not have access to the data and cannot identify who participated in the study.

### Measurement of harmful alcohol consumption

HAC uses AUDIT, a 10‐item screening tool developed by the World Health Organization (WHO).^34^ The reliability and validity of the Japanese version of AUDIT have been confirmed.^32^, ^35^ The Japanese version of AUDIT includes 10 questions about recent alcohol consumption, symptoms of alcohol dependence, and alcohol‐related problems. Each item uses a five‐point Likert scale, and total scores range from 0 to 40. Higher scores indicate a higher level of problematic drinking and an increased risk of hazardous drinking behaviors. Studies have confirmed that AUDIT enables accurate risk assessment across gender, age, and cultural backgrounds.^32^, ^34^ Based on previous studies, we defined the HAC group as those with an AUDIT score of 8 or higher at the second survey, which indicates alcohol use disorders and future harmful risk in this study.^32^, ^33^

### Measurement of occupational stress

Occupational stress was assessed using the General Job Stress Questionnaire (GJSQ), developed by the National Institute for Occupational Safety and Health (NIOSH).^36^ The reliability and validity of the Japanese version of GJSQ have been confirmed.^37^ NIOSH allows independent use of GJSQ subscales to assess occupational stress.^36^ Based on the NIOSH occupational stress model,^36^ this study focused on four subscales: quantitative workload, job control, and social support from coworkers and supervisors. For the job control and social support subscales, higher scores indicate lower stress levels. For quantitative workload, higher scores indicate higher stress levels. Cronbach's alpha coefficients for the sample in this study were 0.669 for quantitative workload, 0.922 for job control, 0.862 for supervisors' support, and 0.861 for coworkers' support.

### Measurement of work–home interaction

The Survey Work–Home Interaction—NijmeGen (SWING), developed in 2005, was used to assess this interaction. Researchers have confirmed the reliability and validity of the Japanese version (SWING‐J).^38^ SWING‐J measures four aspects: negative work‐to‐family spillover (WFNS), negative family‐to‐work spillover (FWNS), positive work‐to‐family spillover (WFPS), and positive family‐to‐work spillover (FWPS). Each item is rated on a four‐point Likert scale from 0 (never) to 3 (always). Researchers calculated total scores for each subscale. Higher scores indicate a greater degree of spillover. Cronbach's alpha coefficients for the sample in this study were 0.879 for WFNS, 0.806 for FWNS, 0.767 for WFPS, and 0.732 for FWPS.

### Demographic variables

Participants provided demographic information, including age, gender (male or female), and marital status (single or married).

### Statistical analysis

Participants were classified into the HAC group (AUDIT score ≥ 8 at the second survey) and the non‐HAC group. Researchers used independent *t*‐tests or chi‐square tests to compare demographic variables, SWING‐J scores, and the four GJSQ subscales between the two groups. The dependent variable was classification into the HAC group. Independent variables included demographic characteristics and scores from the first survey for SWING‐J and GJSQ subscales. A multiple logistic regression analysis was conducted using a backward stepwise procedure. Based on prior literature and theoretical considerations, age, gender, and marital status were forcibly retained in the model as covariates, while other variables were entered and selected through stepwise elimination. Odds ratios (ORs) were calculated to estimate the effects of these variables on being in the HAC group. Statistical significance was set at *p* < 0.05. All analyses used IBM SPSS Statistics version 29.0 (IBM, USA).

## RESULTS

### Participant characteristics

Table 1 summarizes the demographic characteristics, GJSQ scores, SWING‐J scores, and AUDIT scores of the participants. The mean age of the analyzed participants was 46.7 ± 10.4 years. The final analysis included 640 individuals. Of these, 68 (10.6%) were classified in the HAC group, and 572 (89.4%) were in the non‐HAC group. The sample included 451 males (70.5%) and 189 females (29.5%). Among the participants, 242 (37.8%) were unmarried and 398 (62.2%) were married. The mean AUDIT score in the HAC group was 4.8 ± 2.3 at the first survey and 12.5 ± 5.6 at the second survey. An independent samples *t*‐test showed a statistically significant difference in quantitative workload between the HAC and non‐HAC groups. A chi‐square test showed a statistically significant difference in gender distribution between the two groups.

**Table 1:** Participants' characteristics and GJSQ scores and SWING scores in the first survey.

	Range	Total		The HAC group		The non‐HAC group		*p* value
		*n* = 640		*n* = 68 (10.6)		*n* = 572 (89.4)		
		M (SD)	*n* (%)	M (SD)	*n* (%)	M (SD)	*n* (%)	
Age		46.7 (10.4)		49.5 (10.6)		46.4 (10.3)		0.83
Gender								0.023
Male			451 (70.5)		56 (82.4)		395 (69.1)	
Female			189 (29.5)		12 (17.6)		177 (30.9)	
Marital status								0.13
Unmarried			242 (37.8)		20 (29.4)		222 (38.8)	
Married			398 (62.2)		48 (70.6)		350 (61.2)	
GJSQ scores								
Quantitative workload	11–55	34.0 (6.1)		33.3 (4.7)		34.1 (6.2)		0.023
Job control	16–80	46.9 (10.8)		48.4 (10.4)		46.8 (10.9)		0.49
Supervisors support	4–20	12.7 (3.8)		12.8 (3.5)		12.6 (3.9)		0.53
Coworkers support	4–20	13.3 (3.7)		13.3 (3.2)		13.4 (3.7)		0.18
SWING scores								
WFNS	0–24	5.9 (4.8)		7.1 (4.3)		5.8 (4.8)		0.18
FWNS	0–12	2.1 (2.2)		2.6 (2.5)		2.0 (2.2)		0.12
WFPS	0–15	5.4 (3.0)		5.3 (2.5)		5.4 (3.1)		0.062
FWPS	0–15	5.9 (3.1)		5.8 (2.7)		58.7 (3.1)		0.17
AUDIT scores								
The first survey	0–40	2.2 (2.3)		4.8 (2.3)		1.9 (2.1)		0.52
The second survey	0–40	3.0 (4.2)		12.5 (5.6)		1.9 (2.1)		<0.001

*Note*: Values are presented as means ± standard deviations (SD) or frequencies and percentages. *p*‐values represent results of independent *t*‐tests or chi‐square tests comparing problem drinking (AUDIT ≥ 8) and no problem drinking groups.

Abbreviations: AUDIT, alcohol use disorders identification test; FWNS, negative family‐to‐work spillover; FWPS, positive family‐to‐work spillover; GJSQ, the generic job stress questionnaire; HAC, harmful alcohol consumption; SWING, the survey work–home interaction—NijmeGen; WFNS, negative work‐to‐family spillover; WFPS, positive work‐to‐family spillover.

### Logistic regression analysis of the effects of work‐related stress and work–home interaction for HAC

Table 2 presents the results of a multiple logistic regression analysis. This analysis examines the longitudinal risk of HAC in relation to demographic variables, including age, gender, and marital status. It also examines occupational stress measured by GJSQ and work–home interaction measured by SWING‐J.

**Table 2:** Analysis of the risk factors for harmful alcohol consumption (AUDIT ≥ 8 at the second survey) by crude and stepwise multiple logistic regression analysis.

	Crude model			Multiple model		
	OR	(95% CI)	*p*	OR	(95% CI)	*p*
Age	1.03	(0.99–1.06)	0.058			
Gender						
Male	1.00					
Female	0.60	(0.29–1.24)	0.17			
Marital status						
Unmarried	1.00					
Married	1.09	(0.60–1.98)	0.77			
GJSQ scores						
Quantitative workload	0.94	(0.90–0.90)	0.024	0.93	(0.89–0.98)	0.005
Job control	1.02	(0.99–1.04)	0.26			
Supervisors support	1.05	(0.94–1.16)	0.38			
Coworkers support	1.01	(0.90–1.14)	0.84			
SWING scores						
WFNS	1.09	(1.01–1.17)	0.027	1.10	(1.04–1.17)	0.001
FWNS	1.07	(0.93–1.22)	0.38			
WFPS	0.95	(0.83–1.09)	0.47			
FWPS	0.98	(0.86–1.12)	0.76			

*Note*: Independent variables (Multiple model) were entered using backward stepwise logistic regression. Variables that were not statistically significant were removed during model selection.

Abbreviations: AUDIT, alcohol use disorders identification test; CI, confidence interval; FWNS, negative family‐to‐work spillover; FWPS, positive family‐to‐work spillover; GJSQ, the generic job stress questionnaire; OR, odds ratio; SWING, the survey work–home interaction—NijmeGen; WFNS, negative work‐to‐family spillover; WFPS, positive work‐to‐family spillover.

The analysis used stepwise multiple logistic regression. The process began with a full model that included all candidate variables and applied a backward elimination method. In the final model, higher WFNS scores (OR = 1.10, 95% CI = 1.04–1.17) were significantly associated with an increased likelihood of being in the HAC group. In contrast, higher quantitative workload scores (OR = 0.93, 95% CI = 0.89–0.98) were associated with a lower likelihood. The variable selection process excluded other variables from the model.

## DISCUSSION

This study investigated the longitudinal associations between occupational stress, work–family spillover, and HAC over a 6‐month period. The results show that greater WFNS scores were significantly associated with an increased likelihood of being in the HAC group. In contrast, higher quantitative workload was associated with a decreased likelihood. To the best of our knowledge, this is the first longitudinal study to simultaneously examine both the negative and positive dimensions of work–family interactions in relation to HAC.

### Relationship between occupational stress and HAC

Contrary to our hypothesis, our findings suggest that higher quantitative workload was inversely associated with HAC. Numerous studies have established that a combination of high job demands and low job control increases psychological stress and promotes problematic drinking as a coping mechanism.^39^, ^40^ However, our results indicate a different pattern. Some previous studies have reported similar findings. For example, a study among Taiwanese employees found that shorter working hours were associated with a higher risk of alcohol dependence.^41^ Another prospective cohort study reported that perceived job demands had little direct effect on subsequent alcohol consumption.^42^ Taken together, these inconsistencies suggest that the relationship between job demands and HAC may be more complex than previously assumed. One possible explanation is the time‐availability hypothesis. Heavier workloads may reduce the available time or opportunities to engage in drinking behavior. A previous study of middle‐aged workers, aged 40–49, supports this idea and reports a negative association between workload and alcohol use.^15^ Given that the mean age in our sample was 46.8 years, limited drinking opportunities due to time constraints may partially explain our findings.

It is therefore possible that under high workload conditions, individuals may not drink simply because they lack time, even if they experience job stress. Conversely, in the absence of such constraints, individuals may still use alcohol as a coping strategy. Evidence supports this notion, showing that promoting alternative coping mechanisms, such as regular physical exercise, can help reduce alcohol cravings and problematic use in at‐risk individuals.^43^, ^44^ These findings imply that promoting healthy coping strategies, such as physical activity, may represent a promising public health approach to reducing HAC in working populations.

### Relationship between work−family spillover and HAC

Our findings support the hypothesis that WFNS is associated with an increased risk of HAC. A recent cross‐sectional study conducted in Japan reported a positive association between work‐to‐family conflict and problematic drinking.^8^ However, a longitudinal study examining the relationship between work–family conflict and drinking behavior among civil servants found no significant association.^28^ This result was inconsistent with our findings. This discrepancy may be due to differences in sample characteristics. The prior study focused on a single occupational group, specifically civil servants. In contrast, our study included participants from a broader spectrum of occupations, including clerical, technical, nonclerical/nontechnical workers, executives, and the self‐employed. This broader inclusion makes our results potentially more generalizable. Supporting this interpretation, another longitudinal study involving diverse occupations found that men who experienced higher levels of work‐to‐family conflict were more likely to engage in problematic drinking.^29^

Given that 70.9% of participants in our sample were male, WFNS may have a stronger influence on HAC among men. Previous studies also show that work‐related stress contributes to WFNS.^45^ Therefore, in male‐dominated work environments, reducing workplace stress and promoting work–family balance may represent effective strategies to prevent HAC.

### Limitations

This study has several limitations. First, the study sample was limited to Japanese workers, which may restrict the generalizability of the findings to other countries, cultures, or occupational contexts. Second, the sample was predominantly male, and individuals in their 20 s were underrepresented. This may introduce bias and limit the applicability of the results to younger or more gender‐diverse working populations. Third, the relatively modest sample size limited our ability to perform detailed subgroup analyses, such as stratification by age, gender, or occupation, which could have provided deeper insights into differential risk factors for HAC. Fourth, all data were based on self‐reported measures, including alcohol consumption and stress‐related variables. This introduces the possibility of reporting and social desirability bias, which may affect the accuracy of responses. Future longitudinal studies should include larger, more diverse samples and incorporate objective measures when possible to strengthen the validity of findings. Fifth, this two‐wave survey was conducted in 2020 and 2021, during the COVID‐19 pandemic. As such, the results may reflect behavioral and psychological changes specific to that period, particularly in relation to the quantitative workload examined in this study. For instance, factors such as the widespread adoption of remote work, reduced commuting, and temporary reductions in work‐related tasks may have contributed to lower perceived workload during the pandemic.

## CONCLUSION

This longitudinal study examined the associations between HAC, occupational stress, and work‐to‐family and family‐to‐work spillover among Japanese workers. The findings show that higher levels of WFNS may increase the risk of HAC. In contrast, higher quantitative workload was negatively associated with HAC. This negative association may occur because time constraints limit opportunities for alcohol use.

These results highlight the importance of addressing both occupational stress and work–family dynamics in the prevention of HAC. Increasing awareness of stress in both workplace and home environments, along with strategies to support work–life balance, may serve as effective interventions for preventing HAC among working populations.

## AUTHOR CONTRIBUTIONS

*Conceptualization*: Kunio Maekubo and Yasuhiko Deguchi. *Methodology*: Kunio Maekubo and Yasuhiko Deguchi. *Data collection*: Yasuhiko Deguchi and Shinichi Iwasaki. *Data analysis*: Kunio Maekubo and Yasuhiko Deguchi. *Writing—original draft*: Kunio Maekubo. *Writing—review and editing*: Yasuhiko Deguchi, Shinichi Iwasaki, Shohei Okura, and Ayaka Matsunaga: *Supervision*: Koki Inoue. All authors reviewed and approved the final version of the manuscript.

## CONFLICT OF INTEREST STATEMENT

The authors declare no conflicts of interest.

## ETHICS APPROVAL STATEMENT

The Human Subjects Review Committee of Osaka Metropolitan University approved the study protocol (Authorization no. 4245). All data were stored only in our database, and the employer had no access to the data or knowledge of what transpired in the study. Participants were informed that their participation was voluntary.

## PATIENT CONSENT STATEMENT

All participants provided their full informed consent at the top of their questionnaire and were assured that the researchers were not given access to any of their private, identifying information (e.g., name, phone number, and home address) from Macromill, Inc.

## CLINICAL TRIAL REGISTRATION

N/A.

## Data Availability

All data generated or analyzed in this study are included in the published article.
